# Phenotypic Detection of Extended-Spectrum β-Lactamase and Carbapenemase-Producing *Enterobacteriaceae* from Wastewater Treatment Plants in Ouagadougou, Burkina Faso

**DOI:** 10.3390/antibiotics14070641

**Published:** 2025-06-25

**Authors:** Inayatou Hamma Yacouba, Yacouba Konaté, Bapio Valérie Elvira Jean Télesphore Bazie, Boukary Sawadogo

**Affiliations:** 1 Laboratoire Eaux, Hydro-Systèmes et Agriculture (LEHSA), Institut International d’Ingénierie de l’Eau et de L’Environnement (2iE), Ouagadougou 01 BP 594, Burkina Faso; yacouba.konate@2ie-edu.org (Y.K.); boukary.sawadogo@2ie-edu.org (B.S.); 2 Centre National de la Recherche Scientifique et Technologique (CNRST), Institut de Recherche en Sciences de la Santé (IRSS), Ouagadougou 03 BP 7047, Burkina Faso; bazievalery@hotmail.fr

**Keywords:** antibiotic-resistant bacteria, ESBL-E, CPE, wastewater treatment plants, Burkina Faso

## Abstract

**Background/Objectives:** The overuse of antibiotics has led to the emergence of antibiotic-resistant bacteria, posing a global public health challenge. Among these, extended-spectrum β-lactamase and carbapenemase-producing *Enterobacteriaceae* (ESBL-E and CPE) are of particular concern due to their potential to spread resistance in various environments. Understanding the prevalence and spread of these bacteria in the influent and effluent of wastewater treatment plants is essential. **Methods:** This study examined ESBL-E and CPE in wastewater from three WWTPs in Ouagadougou, Burkina Faso, and was conducted between February and August 2024. Phenotypic detection of ESBL-E was performed on the isolates using the double-disk synergy test and the combination disk test, whereas the CPE detection employed the combination disk test and the modified Hodge test. **Results:** A total of 250 *Enterobacteriaceae* isolates were found, with *Escherichia coli*, *Klebsiella* spp., *Enterobacter* spp., and *Buttiauxella* spp. the most predominant taxa. Phenotypic analysis revealed a high prevalence of ESBL-E, particularly in influent samples, with rates ranging from 55 to 98% across the WWTPs. CPE detection showed varying prevalence, with higher proportions identified in effluent samples, ranging from 37 to 68%, depending on the plant. These findings highlight the critical role of WWTPs in the persistence and potential spread of antibiotic-resistant bacteria. **Conclusions:** This study underscores the urgent need for improved wastewater treatment technologies and comprehensive monitoring systems to reduce the dissemination of ESBL-E and CPE in the environment. Addressing these challenges is crucial for mitigating the public health risks associated with antimicrobial resistance.

## 1. Introduction

Antibiotic resistance is a growing global issue that threatens the medical advances achieved over the past decades [1]. This issue has significant implications for infection treatment, disease duration, morbidity, and mortality [2]. These consequences hinder development by delaying progress toward achieving the Sustainable Development Goals (SDGs), particularly those related to health and well-being [3]. In the specific context of Africa, these impacts are exacerbated by limited technical and financial resources and restricted access to healthcare [4,5].

Systematic reviews and meta-analyses have highlighted alarming rates of antimicrobial resistance in enterobacterales infections among children in sub-Saharan Africa, with a particular focus on third-generation cephalosporin resistance and the growing prevalence of carbapenem-resistant strains [6,7]. Among them, *Enterobacteriaceae* are the bacteria found in most environments and have been identified by the World Health Organization (WHO) as critical threats to human health [8,9].

Building on this concern, studies in sub-Saharan Africa, particularly in Burkina Faso, have shown that antibiotic-resistant bacteria are present in various environments across the country, like ready-to-eat foods and clinical settings [10,11]. Beyond these sources, environmental factors also play a crucial role in the dissemination of resistance. In recent years, pharmaceutical compounds have been detected in aquatic environments, with wastewater treatment plants (WWTPs) having a significant impact on the dispersion of these pharmaceutical substances [12,13]. A study by Ouédraogo et al. revealed the presence of antibiotic residues in effluents such as amoxicillin, chloramphenicol, and ceftriaxone discharged into the environment, raising concerns not only about the potential toxicity of these compounds to aquatic organisms and humans but also because of the emergence of resistance in pathogenic bacteria [14].

The presence of extended-spectrum β-lactamase-producing *Enterobacteriaceae* (ESBL-E) in wastewater represents a major public health issue, as these strains can be transmitted through environmental pathways [15,16]. Carbapenemase-producing *Enterobacteriaceae* (CPE) are particularly concerning due to their resistance to carbapenems, which are commonly used as last-line antibiotics for treating serious infections [17].

In this context, Burkina Faso is deeply affected by the challenges posed by rapid urbanization and population growth, particularly in managing water resources and wastewater [18]. Effective wastewater management is crucial for protecting the environment and public health, especially in rapidly expanding urban areas such as Ouagadougou.

However, current knowledge about antibiotic-resistant bacteria in wastewater from wastewater treatment plants (WWTPs) in Burkina Faso remains very limited, most studies focus on clinical settings. In response to this gap, research has been conducted to provide a deeper understanding of the antibiotic resistance mechanisms of bacteria isolated from WWTPs. The lack of data on the prevalence of antibiotic-resistant bacteria in wastewater treatment plants in Burkina Faso justifies this study, which aims to phenotypically detect and assess the prevalence of ESBL-E and CPE. This study will also help evaluate the effectiveness of current treatment processes in eliminating these resistant microorganisms.

## 2. Results

### 2.1. Bacterial Isolation

**Figure 1:**
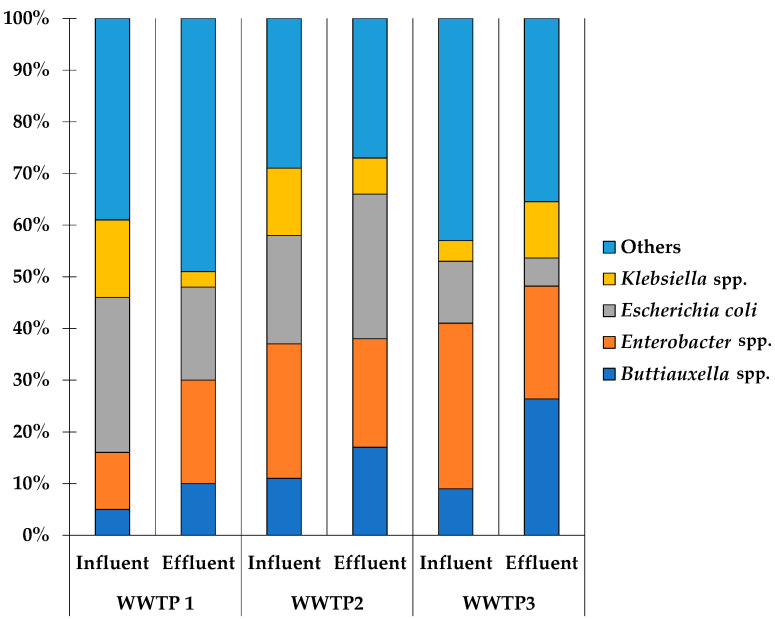
Proportion of predominant taxa identified in three WWTPs.

The total number of *Enterobacteriaceae* isolates found across the three wastewater treatment plants (WWTPs) was 250. In WWTP 1, isolates were identified in 40% (101/250) of the samples, with 61/101 (60%) from influent samples and 40/101 (40%) from effluent samples. In WWTP 2, isolates were found in 30% (76/250) of the samples, comprising 47/76 (62%) in influent samples and 29/76 (38%) in effluent samples. In WWTP 3, isolates were detected in 29% (73/250) of the samples, with 57/73 (78%) from influent samples and 16/73 (22%) from effluent samples. The most predominant taxa were *Escherichia coli*, followed by *Klebsiella* spp., *Enterobacter* spp., and *Buttiauxella* spp., as shown in Figure 1 and Table S1.

### 2.2. Phenotypic Detection from Three WWTPs

Among the 250 *Enterobacteriaceae* isolates analyzed, phenotypic detection identified 167 (67%) as ESBL-E. From WWTP 1, 61/101 (60%) isolates were ESBL-E-positive, with a distribution of 67% (41/61) in influent samples and 50% (20/40) in effluent samples. In WWTP 2, 38/76 (50%) isolates were positive, comprising 55% (26/47) of the influent samples and 41% (12/29) of the effluent samples. In WWTP 3, 68/73 (93%) isolates were ESBL-E-positive, with 98% (56/57) in the influent samples and 75% (12/16) in the effluent samples.

Similarly, phenotypic detection identified 109 (44%) isolates as CPE. In WWTP 1, 52/101 (51%) isolates were positive, including 41% (25/61) in the influent samples and 68% (27/40) in the effluent samples. WWTP 2 recorded 30/76 (39%) positive isolates, with 32% (15/47) in the influent samples and 52% (15/29) in the effluent samples. In WWTP 3, 27/73 (37%) isolates were CPE-positive, with 37% (21/57) in influent samples and 38% (6/16) in effluent samples.

**Figure 2:**
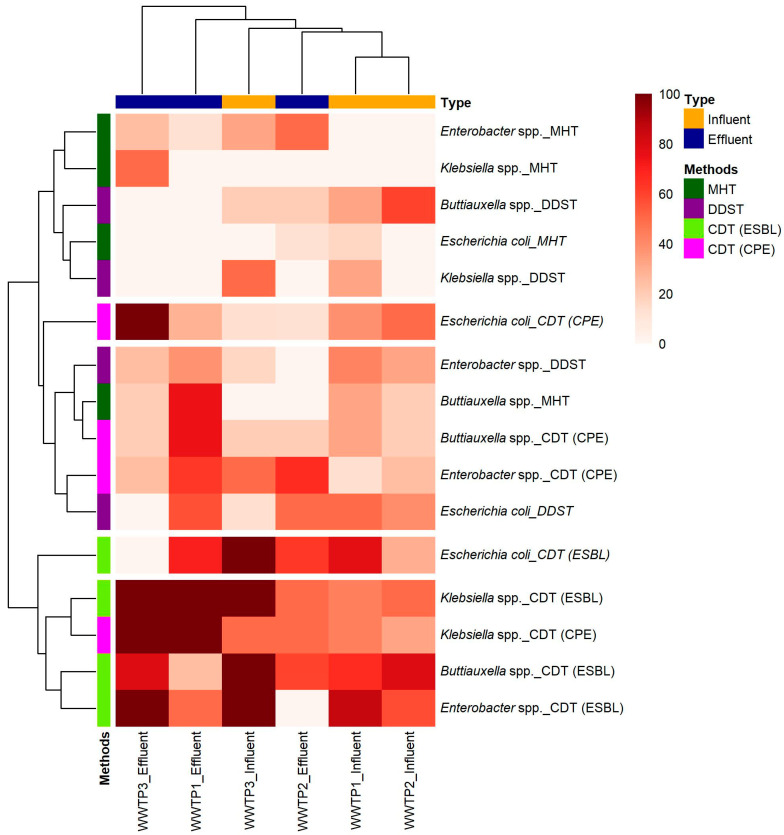
Heatmap of phenotypic detection of ESBL-E and CPE among predominant bacterial taxa across three WWTPs, based on comparative methodological approaches.

An overview of the different phenotypic techniques used for detecting ESBL-E and CPE, such as the double-disk synergy test (DDST), the combination disk test (CDT), and the modified Hodge test (MHT), alongside the predominant taxa identified during the study, is given in Figure 2 and Table S2. This highlights the variability in detection rates across taxa (*Buttiauxella* spp., *Enterobacter* spp., *Escherichia coli*, and *Klebsiella* spp.) and between influent and effluent samples across the three WWTPs.

### 2.3. Co-Occurrence of ESBL-E and CPE

The phenotypic detection revealed the co-occurrence of ESBL-E and CPE within various taxa, including *Buttiauxella* spp., *Enterobacter* spp., *E. coli*, and *Klebsiella* spp.

Across the WWTPs, *Enterobacter* spp. were more prevalent in the influent samples, especially in WWTP 3. *Escherichia coli* was mostly found in influents, while *Klebsiella* spp. were detected in both influents and effluents, often with higher prevalence in effluents. *Buttiauxella* spp. were found to be inconsistently distributed, appearing in the effluent of WWTP 1 and only in the influent of WWTP 3.

These findings are summarized in Table 1, which highlights the distribution of taxa positive for both ESBL-E and CPE in influent and effluent samples across the three WWTPs.

### 2.4. Determination of the Antimicrobial Susceptibility Pattern of Enterobacteriaceae

The resistance patterns of ESBL-E and CPE to various antibiotics across the three wastewater treatment plants (WWTPs) revealed significant trends, highlighting antibiotics with both high and low resistance rates.

For ESBL-E, high resistance was observed for ampicillin (100% in WWTP 1 influent, and both influent and effluent of WWTP 3), ceftriaxone (ranging from 70 to 100%), cefepime (80–90% across all WWTPs), and imipenem (90–100% in all WWTPs). Conversely, low resistance rates were consistently noted for doripenem (0–20%), MRP (0–20%), and piperacillin-tazobactam (0–20%) across both the influent and effluent samples (Table 2).

Similarly, the CPE exhibited comparable resistance trends. High resistance percentages were recorded for AMP (70–100% across all the WWTPs), CTR (50–100%), FEP (50–100%), and IMP (90–100%). In contrast, resistance to DOR (10–30%), MRP (10–30%), and TZP (10–30%) remained low in both the influent and effluent samples (Table 3).

### 2.5. Multidrug Resistance

During our study, the ESBL and CPE strains demonstrated resistance to more than three antibiotics (Table 4). These findings highlight variations in multiresistance levels among different wastewater treatment plants (WWTPs).

## 3. Discussion

The emergence and dissemination of antimicrobial resistance (AMR) have become significant global public health concerns, exacerbated by the overuse and misuse of antibiotics in human, veterinary, and agricultural settings, as well as the discharge of untreated or inadequately treated wastewater into the environment [19,20]. Wastewater treatment plants (WWTPs) serve as both reservoirs and dissemination points for antimicrobial-resistant bacteria (ARB) and antimicrobial resistance genes (ARGs) [21]. The release of these resistant bacteria into receiving waters can facilitate the persistence and spread of AMR within aquatic ecosystems, posing risks to human health and environmental microbial communities [22].

### 3.1. Bacterial Isolation

In our study, the predominant species identified were *Escherichia coli*, *Klebsiella* spp., *Buttiauxella* spp., and *Enterobacter* spp. Similarly, a recent study by Urzua-Abad et al. characterized bacterial species isolated from hospital and municipal wastewater in Mexico City, describing several enteric and non-enteric Gram-negative strains [23]. Another study by Nasser-Ali et al. also found human and environmental bacteria in wastewater from urban wastewater treatment plants in Coruña, Spain [24]. Regarding *Buttiauxella* spp., this species is more commonly found in environmental settings than in humans [25]. However, another study discovered *Buttiauxella agrestis* present in sewage sludge [26].

### 3.2. Phenotypic Detection

The overall prevalence of ESBL-E among *Enterobacteriaceae* isolates in our study (67%) is comparable to studies that reported high ESBL rates in wastewater environments [27]. Our results demonstrate that WWTP 3 had the highest ESBL-E prevalence (93%), particularly in the influent samples (98%). This aligns with a study in which ESBL-producing bacteria were detected in 97.6% of wastewater samples collected from five healthcare centers in Burkina Faso [28]. This highlights the range of antibiotic-resistant bacteria in hospital effluents, often exceeding those found in municipal sewage systems [13,29]. The reduction observed in the effluent samples (75%) aligns with findings from other studies indicating incomplete removal of resistant bacteria during wastewater treatment [20]. However, the persistence of such a high proportion in effluent underscores the limitations of conventional treatment processes in fully eliminating resistant pathogens.

The CPE prevalence in this study (44%) is slightly higher than what some studies have reported but is within the range documented in regions with high antimicrobial usage [27]. Our findings from WWTP 1 (51% CPE-positive isolates) and the notable increase in effluent samples (68%) reflect trends observed in studies suggesting that selective pressure during treatment processes may favor the survival of carbapenemase producers [30]. We can also hypothesize horizontal gene transfer, as well as the insufficient efficiency of wastewater treatment processes [20,24,31]. In comparison, WWTP 3 recorded the lowest CPE prevalence (37%), with minimal variation between the influent and effluent samples, indicating possible differences in influent composition. Industrial processes may contribute more significantly to the prevalence of these resistance genes, as highlighted by Wu et al., where the discharge of heavy metals or other pollutants from industrial activities can be responsible for certain resistance traits [32]. Additionally, the types and quantities of antibiotics consumed by the general population, as well as their subsequent excretion into sewage systems, contribute substantially to shaping the resistance profiles observed in wastewater [33].

### 3.3. Comparison of Techniques and Taxa Prevalence

The CDT technique demonstrated greater sensitivity for detecting ESBL-E compared with DDST (Figure 2). DDST, which is useful for detecting ESBLs, may be less sensitive in certain contexts [34]. Therefore, CDT is generally more sensitive and can detect a broader range of resistance mechanisms, making it effective for identifying ESBL-E in wastewater samples [35]. A study in India by Sageerabanoo et al. highlighted the superiority of CDT over other methods for ESBL detection, which is consistent with our findings [36].

MHT showed a slightly lower sensitivity than CDT in detecting CPE (Figure 2). However, MHT is particularly useful for identifying CPE but may not provide information on some resistance mechanisms detected by CDT, such as metallo-beta-lactamase (MBL) [37]. Kumudunie et al. emphasized the limitations of MHT, where MHT could detect only 50% of NDM-producing strains, which corroborates our observation that CDT detected a higher proportion of CPE across all taxa [38].

### 3.4. Dual Resistance

The dominance of *Enterobacter* spp. and *Klebsiella* spp. as carriers of both ESBL-E and CPE is consistent with findings in prior studies, such as those by Garba et al. and Haller et al., which highlighted these taxa as significant reservoirs of multidrug resistance in wastewater [28,39]. The persistence of *Klebsiella* spp. in effluents aligns with observations of their resilience and ability to survive standard treatment processes, such as *Klebsiella pneumonia* ST101, which can withstand chlorine treatment [40]. *Escherichia coli*, while frequently detected as ESBL-E, is less commonly found with dual resistance, reflecting its lower environmental resilience compared to *Klebsiella* spp. [41,42]. The limited data on *Buttiauxella* spp. in the literature make direct comparisons challenging, but its emerging presence in wastewater, as noted in this study, needs further investigation into its role in AMR dissemination.

### 3.5. Antibiotic Susceptibility

The widespread resistance to AMP (70–100%) observed in both the ESBL-E and CPE isolates aligns with its frequent use in human and veterinary medicine [43]. A study by Belachew et al. similarly reported high AMP resistance rates, emphasizing the selective pressure exerted by its extensive use [44]. Previous research in West Africa, including studies in Burkina Faso, has identified similar resistance trends in environmental and clinical isolates, underscoring the regional significance of penicillin resistance [5].

Resistance to CTR (70–100%) and FEP (80–90%) highlights the pervasive impact of third-generation cephalosporins on the selection of resistant strains. Notably, ceftriaxone residues were detected in WWTP 1, highlighting the role of wastewater treatment plants as reservoirs for these antibiotics and their contributions to resistance selection [14]. These findings are consistent with studies that link environmental contamination by healthcare and agricultural effluents to increased resistance in aquatic ecosystems [45,46].

Resistance to IMP (90–100%) among CPE isolates is a significant concern. The high resistance levels observed in this study mirror findings from similar wastewater studies globally, where carbapenem-resistant *Enterobacteriaceae* (CRE) are increasingly reported [47]. This resistance compromises the efficacy of last-resort antibiotics and aligns with studies that have documented carbapenem resistance in treated effluents, raising questions about the effectiveness of current wastewater treatment processes [48].

Conversely, resistance to DOR, MRP, and TZP (0–30%) remained low, indicating that these antibiotics are limited in terms of usage and reduce selective pressure in the studied environments. This can be explained by a 2022 study on the use of WATCH antibiotics prior to hospital admission in rural Burkina Faso, which revealed that ceftriaxone, a WATCH category antibiotic, was used significantly more than carbapenems in hospital settings [49]. The low resistance rates provide optimism for their continued use in combating resistant infections.

### 3.6. Multidrug Resistance

These findings from Table 4 are consistent with studies from wastewater environments worldwide, where ESBL-E isolates frequently exhibit MDR, due to multiple resistance mechanisms and the presence of plasmids and mobile genetic elements [35,50]. The results in Table 4 also align with global studies reporting that carbapenem-resistant strains are often MDR, driven by plasmid-mediated resistance mechanisms such as carbapenemase genes and additional resistance determinants [51,52,53]. Moreover, the high rates of MDR observed in WWTP 3 suggest significant selective pressure from healthcare effluents, as reported in studies on hospital wastewater systems [13,28,29].

### 3.7. Limitations of the Study

In this study, we evaluated the performance of the combination disk test and double disk synergy test for detecting extended-spectrum β-lactamases-producing *Enterobacteriaceae* and the combination disk test and modified Hodge test for detecting carbapenemase-producing *Enterobacteriaceae*. However, we did not employ molecular methods as reference standards to confirm the presence of extended-spectrum β-lactamase or carbapenemase genes. This could potentially affect the accuracy of our phenotypic detection results, as molecular methods are widely regarded as the gold standard for such analyses. Future studies should incorporate molecular techniques to validate and enhance the reliability of phenotypic detection methods.

## 4. Materials and Methods

### 4.1. Sites and Sampling

The study area is in West Africa, in Ouagadougou, the capital of Burkina Faso. This study focuses on three wastewater stabilization ponds serving as wastewater treatment plants in the city of Ouagadougou, Burkina Faso. Most of the raw urban wastewater treated by the first WWTP, the Kossodo treatment plant, comes from various sources, including domestic and hospital wastewater as well as pre-treated (settled) industrial wastewater from the slaughterhouse and the brewery industry, the latter being a major contributor. Only domestic wastewater is treated at the second WWTP, the 2iE Institute Treatment Plant. The third WWTP, the Tengandogo University Hospital Treatment Plant, treats only wastewater from the hospital. Monthly sampling was carried out at each WWTP between February and August 2024. All the WWTPs’ wastewater samples were taken instantaneously at the effluent and as 24 h composites at the influent. A total of 38 liquid effluent samples were collected, 14 from WWTP 1 and 12 from WWTP 1 and WWTP 3. The samples were transported to the laboratory, kept at 4 °C, and processed immediately.

### 4.2. Bacterial Isolation

After transporting the samples from the treatment plants, tenfold serial dilutions (10–1000 times) of each water sample were made in sterile saline solution. A total of 1 mL of each dilution was then plated onto MacConkey agar supplemented with cefotaxime (1 μg/mL) or meropenem (0.25 μg/mL). After incubation for 24–48 h at 35 ± 2 °C, bacterial colonies with distinct coloration and morphologies were randomly picked and subcultured onto MacConkey agar containing cefotaxime (1 μg/mL) or meropenem (0.25 μg/mL) for further purification. A single colony of purified bacteria was picked and cultured in Trypticase Soy Broth. Each isolate was assigned a unique identification number and stored at −80 °C in 20% (*v*/*v*) glycerol for further investigation.

### 4.3. Identification

The pure isolates were screened for Gram-negative bacteria using a chemical method, the potassium hydroxide (KOH) test [54]. The isolates were subcultured on Eosin Methylene Blue (EMB) agar and Salmonella-Shigella (SS) agar. The plates were incubated at 37 °C for 24 h. Species-level identification of the isolates was performed using standard biochemical test protocols (Table S3). The Gram-negative isolates were tested for catalase activity, motility, indole production, methyl red reaction, Voges-Proskauer reaction, citrate utilization, urease activity, hydrogen sulfide, acid, and gas production in Triple Sugar Iron (TSI) agar. The results were interpreted following Bergey’s Manual of Systematics of Archaea and Bacteria [55].

### 4.4. Antibiotic Susceptibility Test

Antibiotic susceptibility testing was conducted on the *Enterobacteriaceae* isolates using twelve antibiotics, including four cephalosporins (cefotaxime 30 µg, ceftriaxone 30 µg, cefepime 30 µg, and ceftazidime 30 µg), four carbapenems (imipenem 10 µg, ertapenem 10 µg, meropenem 10 µg, and doripenem 10 µg), and other antibiotics such as ampicillin 10 µg, amoxicillin-clavulanate 20 µg/10 µg, aztreonam 30 µg, and piperacillin-tazobactam 100 µg/10 µg. The disc diffusion method, as described by Bauer et al., was employed to assess antibiotic susceptibility [56]. A single colony from an overnight bacterial culture was suspended in sterile saline solution and adjusted to a turbidity of 0.5 McFarland. Sterile Mueller-Hinton agar (MHA) plates were inoculated with the bacterial suspension, spread evenly using a sterile swab, and allowed to dry. Antibiotic discs were placed on the agar surface using sterile forceps. The plates were then incubated at 35 °C ± 2 °C for 18–24 h. Following incubation, the diameter of the inhibition zones was measured and interpreted according to the Antibiogram Committee of the French Society for Microbiology in association with the European Committee on Antimicrobial Susceptibility Testing (CA-SFM/EUCAST) [57,58].

### 4.5. Phenotypic Detection of β-Lactamases

#### 4.5.1. Screening Enterobacteriaceae for ESBL Production

The double disk synergy test (DDST) was employed to confirm the presence of extended-spectrum β-lactamases (ESBL) production in bacterial isolates resistant to cephalosporins and penicillin, following CA-SFM/EUCAST guidelines [57]. The bacterial isolates were adjusted to 0.5 McFarland turbidity standards and evenly inoculated onto Mueller-Hinton agar (MHA) plates. Antibiotic disks containing aztreonam (30 µg), cefepime (30 µg), ceftriaxone (30 µg), ceftazidime (30 µg), and cefotaxime (30 µg) were positioned 30 mm (center to center) from a central amoxicillin-clavulanate (20 µg/10 µg) disk. The plates were incubated aerobically at 37 °C for 18–24 h. Following incubation, the isolates were evaluated for ESBL production. A clear extension of the inhibition zone of any cephalosporin disk toward the central amoxicillin-clavulanate disk, with an observed increase in the zone diameter of at least 5 mm compared with that of the cephalosporin disk alone, was interpreted as indicative of ESBL production.

Following the Clinical and Laboratory Standards Institute (CLSI) guidelines, the combination disk test (CDT) was employed to confirm the ESBL production [59]. Ceftazidime (30 µg) and cefotaxime (30 µg) disks, both with and without clavulanic acid, were placed at appropriate distances on a Muller-Hinton agar (MHA) plate inoculated with a bacterial suspension standardized to 0.5 McFarland turbidity. The plates were incubated aerobically at 37 °C for 16–18 h. An isolate was confirmed as an ESBL producer if the inhibition zone diameter around the combination disk (antibiotic plus clavulanic acid) increased by more than 5 mm compared with the inhibition zone around the antibiotic disk alone.

#### 4.5.2. Screening Enterobacteriaceae for Carbapenemase Production

The combination disk test (CDT) used by Chauhan et al. was performed to confirm carbapenemase production [60]. Imipenem (10 µg) disks, with and without ethylenediaminetetraacetic acid (EDTA), were placed at a standardized distance on Mueller-Hinton agar (MHA) plates inoculated with a bacterial suspension adjusted to 0.5 McFarland turbidity. The plates were incubated aerobically at 37 °C for 16–18 h. Carbapenemase production was confirmed if the inhibition zone diameter around the combination disk (imipenem plus EDTA) was at least 7 mm larger than the inhibition zone around the imipenem disk alone.

The modified Hodge test (MHT) was conducted to detect carbapenemase production following the CLSI guidelines [61]. A suspension of *E. coli* ATCC 25922 was prepared to a 0.5 McFarland standard and uniformly swabbed onto MHA plates. After the plate was allowed to dry, a 10 μg ertapenem disk was placed at the center, and the test isolates were streaked in a straight line from the edge of the disk to the periphery of the plate. The plates were incubated at 37 °C for 16–18 h. The presence of a cloverleaf-like indentation in the inhibition zone around the ertapenem disk, near the growth of *E. coli* ATCC 25922, was interpreted as a positive result for carbapenemase production.

### 4.6. Quality Control

The quality of the media, reagents, and antibiotic discs was ensured by strictly following the manufacturer’s instructions (Liofilchem, Roseto degli Abruzzi, Italy). To verify the reliability of the antibiotic susceptibility tests, phenotypic detection methods, and biochemical assays, the reference strain *Escherichia coli* ATCC 25922 was employed as a quality control.

### 4.7. Statistical Analysis

The data were entered, cleaned, and analyzed using Microsoft Excel (version 2024). Descriptive statistics were used to summarize the findings. The prevalence of ESBL-E and CPE, based on comparative methodological approaches, was visualized using a heatmap generated with the pheatmap package in R (RStudio, version 4.5.0).

## 5. Conclusions

This study highlights the prevalence of extended-spectrum β-lactamase and carbapenemase-producing *Enterobacteriaceae* in wastewater treatment plants in Ouagadougou, Burkina Faso. The high levels of ESBL strains show the increasing difficulty of treating infections caused by bacteria resistant to common antibiotics, whereas the detection of carbapenemase-producing bacteria signals the growing threat of resistance to last-resort antibiotics. These findings underscore the important contribution of wastewater treatment plants to the spread of antibiotic resistance in the environment, emphasizing the urgent need to upgrade treatment infrastructure through advanced methods such as membrane filtration or UV disinfection, which can improve the removal of resistant bacteria and genes. Moreover, implementing routine surveillance programs for resistant strains in wastewater systems will facilitate early detection and targeted interventions to mitigate environmental dissemination. This research calls for improved wastewater management and public health efforts, involving national health authorities, environmental agencies, municipalities, and wastewater treatment plant operators, in order to resolve the rising problem of antibiotic resistance and protect both human and environmental health.

## Figures and Tables

**Table 1:** Co-occurrence of ESBL-E and CPE in predominant taxa from three WWTPs.

WWTPs	ESBL-E and CPE Strains N (%)							
	*Buttiauxella* spp.		*Enterobacter* spp.		*Escherichia Coli*		*Klebsiella* spp.	
	Influent	Effluent	Influent	Effluent	Influent	Effluent	Influent	Effluent
WWTP 1	0/3 (0)	1/4 (25)	1/7 (14)	4/8 (50)	4/18 (22)	1/7 (14)	1/9 (11)	1/1 (100)
WWTP 2	1/5 (20)	1/5 (20)	3/12 (25)	0/6 (0)	3/10 (30)	1/8 (13)	1/6 (17)	1/2 (50)
WWTP 3	1/5 (20)	0/5 (0)	11/18 (61)	1/4 (25)	1/7 (14)	1/1 (100)	1/2 (50)	2/2 (100)

**Table 2:** Overall percentage resistance of ESBL-E to different antibiotics.

Antibiotics	WWTP 1		WWTP 2		WWTP 3	
	Influent	Effluent	Influent	Effluent	Influent	Effluent
AMP	100	90	80	80	100	100
ATM	70	70	60	70	90	80
AUG	70	50	50	50	80	100
CAZ	80	70	60	50	90	70
CTR	90	90	80	70	100	80
CTX	80	80	60	60	90	80
DOR	0	10	0	10	20	20
ETP	20	30	30	20	40	70
FEP	90	90	80	80	90	80
IMP	90	100	90	90	90	100
MRP	0	10	0	10	10	20
TZP	10	10	0	20	20	0

AMP = ampicillin, ATM = aztreonam, AUG = amoxicillin/clavulanic acid, CAZ = ceftazidime, CTR = ceftriaxone, CTX = cefotaxime, DOR = doripenem, FEP = cefepime, IMP = imipenem, MRP = meropenem, TZP = piperacillin-tazobactam.

**Table 3:** Overall percentage resistance of CPE to different antibiotics.

Antibiotics	WWTP 1		WWTP 2		WWTP 3	
	Influent	Effluent	Influent	Effluent	Influent	Effluent
AMP	80	80	70	70	100	100
ATM	60	40	30	30	90	70
AUG	60	60	50	60	70	80
CAZ	70	40	40	20	90	80
CTR	80	60	60	50	100	70
CTX	70	60	40	30	100	70
DOR	20	20	10	10	30	30
ETP	50	40	30	50	40	70
FEP	80	60	50	50	100	70
IMP	90	100	90	90	100	100
MRP	20	30	10	10	20	30
TZP	20	20	10	10	30	20

AMP = ampicillin, ATM = aztreonam, AUG = amoxicillin/clavulanic acid, CAZ = ceftazidime, CTR = ceftriaxone, CTX = cefotaxime, DOR = doripenem, FEP = cefepime, IMP = imipenem, MRP = meropenem, TZP = piperacillin-tazobactam.

**Table 4:** Multidrug resistance profiles of ESBL-E and CPE from three WWTPs.

Strains	WWTP 1		WWTP 2		WWTP 3	
	Influent N (%)	Effluent N (%)	Influent N (%)	Effluent N (%)	Influent N (%)	Effluent N (%)
ESBL-E	40/41 (98)	19/20 (95)	22/26 (85)	9/12 (82)	56/56 (100)	12/12 (100)
CPE	22/25 (88)	23/27 (85)	10/15 (67)	11/15 (73)	21/21 (100)	6/6 (100)

## Data Availability

The data of this study are available on request from the authors.

## Supplementary Materials

The following supporting information can be downloaded at: https://www.mdpi.com/article/10.3390/antibiotics14070641/s1, Table S1. Distribution of predominant taxa identified across the Three WWTPs. Table S2. Phenotypic detection of predominant taxa from three WWTPs. Table S3. Biochemical profile of representative species from predominant bacterial taxa isolated from WWTPs.
