# Supplementary material for: Phenotypic Detection of Extended-Spectrum β-Lactamase and Carbapenemase-Producing Enterobacteriaceae from Wastewater Treatment Plants in Ouagadougou, Burkina Faso
Source: Antibiotics (Basel). 2025 Jun 25;14(7):641. doi: 10.3390/antibiotics14070641 (PMC12291763; doi:10.3390/antibiotics14070641)
Supplement: Supplementary file 1 [file antibiotics-14-00641-s001.zip › antibiotics-3638484-supplementary.pdf]

Table S1. Distribution of predominant taxa identified across the Three WWTPs.

WWTPs	*Buttiauxella* spp.		*Enterobacter* spp.		*Escherichia coli*		*Klebsiella* spp.	
	Influent N (%)	Effluent N (%)	Influent N (%)	Effluent N (%)	Influent N (%)	Effluent N (%)	Influent N (%)	Effluent N (%)
WWTP 1	3/61 (5)	4/40 (10)	7/61 (11)	8/40 (20)	18/61 (30)	7/40 (18)	9/61 (15)	1/40 (3)
WWTP 2	5/47 (11)	5/29 (17)	12/47 (26)	6/29 (21)	10/47 (21)	8/29 (28)	6/47 (13)	2/29 (7)
WWTP 3	5/57 (9)	5/17 (29)	18/57 (32)	4/17 (24)	7/57 (12)	1/17 (6)	2/57 (4)	2/17 (12)

Table S2. Phenotypic detection of predominant taxa from three WWTPs.

Taxa	WWTPs	ESBL-E strains N (%)				CPE strains N (%)			
		DDST		CDT		MHT		CDT	
		Influent	Effluent	Influent	Effluent	Influent	Effluent	Influent	Effluent
Buttiauxella spp.	1	1 (33)	0 (0)	2 (67)	1 (25)	1 (33)	3 (75)	1 (33)	3 (75)
	2	3 (60)	1 (20)	4 (80)	3 (60)	1 (20)	0 (0)	1 (20)	1 (20)
	3	1 (20)	0 (0)	5 (100)	4 (80)	0 (0)	1 (20)	1 (20)	1 (20)
Enterobacter spp.	1	3 (43)	3 (38)	6 (86)	4 (50)	0 (0)	1 (13)	1 (14)	5 (63)
	2	4 (33)	0 (0)	7 (58)	0 (0)	0 (0)	3 (50)	3 (25)	4 (67)
	3	3 (17)	1 (25)	18 (100)	4 (100)	6 (33)	1 (25)	9 (50)	1 (25)
Escherichia Coli	1	9 (50)	4 (57)	14 (78)	5 (71)	3 (17)	0 (0)	7 (39)	2 (29)
	2	4 (40)	4 (50)	3 (30)	5 (63)	0 (0)	1 (13)	5 (50)	1 (13)
	3	1 (14)	0 (0)	7 (100)	0 (0)	0 (0)	0 (0)	1 (14)	1 (100)
Klebsiella spp.	1	3 (33)	0 (0)	4 (44)	1 (100)	0 (0)	0 (0)	4 (44)	1 (100)
	2	0 (0)	0 (0)	3 (50)	1 (50)	0 (0)	0 (0)	2 (33)	1 (50)
	3	1 (50)	0 (0)	2 (100)	2 (100)	0 (0)	1 (50)	1 (50)	2 (100)

Table S3. Biochemical profile of representative species from predominant bacterial taxa isolated from WWTPs.

						TSI								
Code	catalase	MR	VP	Motility	Indole	H_2S	Slant/Butt	Gas	Citrate	Urea	MacConkey agar	SS agar	EMB agar	Species
**MB07IM**	**+**	**+**	**-**	**+**	**+**	**-**	**A/A**	**+**	**-**	**-**	**Pink**	**Pink**	**metallic green sheen**	***Escherichia coli***
**CB01IM**	**+**	**+**	**-**	**-**	**-**	**-**	**A/A**	**+**	**-**	**-**	**Pink**	**Pink**	**Pink**	***Klebsiella pneumoniae***
**CB02TMM**	**+**	**+**	**-**	**+**	**-**	**-**	**A/A**	**+**	**-**	**-**	**Pink**	**Colorless**	**Pink**	***Buttiauxella izardii***
**CB01KA**	**+**	**+**	**-**	**-**	**-**	**-**	**A/A**	**+**	**+**	**-**	**Pink**	**-**	**Pink**	***Enterobacter asburiae***

A = Acidic
